# Fast Feature- and Category-Related Parafoveal Previewing Support Free Visual Exploration

**DOI:** 10.1523/JNEUROSCI.0841-24.2024

**Published:** 2024-10-25

**Authors:** Camille Fakche, Clayton Hickey, Ole Jensen

**Affiliations:** ^1^Centre for Human Brain Health, School of Psychology, University of Birmingham, Birmingham B15 2TT, United Kingdom; ^2^Department of Experimental Psychology, University of Oxford, Oxford OX2 6GG, United Kingdom; ^3^Oxford Centre for Human Brain Activity, Wellcome Centre for Integrative Neuroimaging, Department of Psychiatry, University of Oxford, Oxford OX3 7JX, United Kingdom

**Keywords:** classification, foveal processing, free visual exploration, object categorization, parafoveal processing, pipelining mechanism

## Abstract

While humans typically saccade every ∼250 ms in natural settings, studies on vision tend to prevent or restrict eye movements. As it takes ∼50 ms to initiate and execute a saccade, this leaves only ∼200 ms to identify the fixated object and select the next saccade goal. How much detail can be derived about parafoveal objects in this short time interval, during which foveal processing and saccade planning both occur? Here, we had male and female human participants freely explore a set of natural images while we recorded magnetoencephalography and eye movements. Using multivariate pattern analysis, we demonstrate that future parafoveal images could be decoded at the feature and category level with peak decoding at ∼110 and ∼165 ms, respectively, while the decoding of fixated objects at the feature and category level peaked at ∼100 and ∼145 ms. The decoding of features and categories was contingent on the objects being saccade goals. In sum, we provide insight on the neuronal mechanism of presaccadic attention by demonstrating that feature- and category-specific information of foveal and parafoveal objects can be extracted in succession within a ∼200 ms intersaccadic interval. These findings rule out strict serial or parallel processing accounts but are consistent with a pipeline mechanism in which foveal and parafoveal objects are processed in parallel but at different levels in the visual hierarchy.

## Significance Statement

We provide neural evidence that future parafoveal saccade goals are processed surprisingly quickly at the feature and the category level before we saccade to them. Specifically, using multivariate pattern analysis applied to magnetoencephalography and eye-tracking data, we found that information about the color and the category of parafoveal objects emerged at ∼110 and ∼165 ms, respectively, with the same information about foveal objects emerging ∼100 and ∼145 ms. Our findings provide novel insight into the neuronal dynamics of parafoveal previewing during free visual exploration. The dynamics rule out strict serial or parallel processing but are consistent with a pipelining mechanism in which foveal and parafoveal objects are processed in parallel but at different levels in the visual hierarchy.

## Introduction

Humans have a remarkable ability to explore visual scenes efficiently. This capacity relies on eye movements occurring every ∼250 ms, which shifts the fovea to informative parts of the visual scene (Yarbus, 1967; Prasad and Galetta, 2011). Considering that the oculomotor system takes ∼50 ms to initiate and execute a saccade, the visual system has only ∼200 ms to identify the fixated object and select the next saccade goal if processing is entirely serial (Otero-Millan et al., 2008). This seems implausible and has led to the proposal that visual cognition involves parallel processing. For example, pipelining theory (Jensen et al., 2021) suggests that foveal and parafoveal objects can be processed simultaneously but at different levels of the visual hierarchy. Meanwhile, the planning of the next saccade occurs in the oculomotor areas. Our study aims to identify the neuronal dynamics of parafoveal (2–5° eccentricity) processing that occurs during the ∼200 ms intersaccadic interval during free visual exploration, in parallel with the foveal processing and the saccade preparation.

Parafoveal processing has three likely functions. First is to support the preparation of saccade goals. Since our eyes typically land on informative parts of visual scenes, a selection must be made between saccade goals. Studies using eye-tracking have provided evidence for parafoveal processing at semantic and category level guiding saccades during visual search (Henderson et al., 1999; Bonitz and Gordon, 2008; LaPointe and Milliken, 2016; Nuthmann et al., 2019; Borges et al., 2020; Cimminella et al., 2020). An electroencephalography (EEG) study has found electrophysiological support for semantic parafoveal previewing during natural scene viewing, i.e., the category of the parafoveal object did not match with the natural scene (Coco et al., 2020). However, the effect was observed at ∼400 ms and was therefore too slow to impact the next saccade plan. The first aim of our study was to investigate whether parafoveal previewing of categories of visual objects was identifiable in the brain data in the intersaccadic interval.

The second function of parafoveal previewing is to give visual processing a head start. Parafoveal previewing serves to speed up the processing of the object when fixated, i.e., the preview benefit (Rayner, 1998; Huber-Huber et al., 2021). Paradigms relying on controlled eye movements showed that the performance was faster (∼30–150 ms) when an object were previewed (Henderson et al., 1987, 1989, Pollatsek et al., 1990; Henderson, 1992) and that the detection performance of low- and high-level visual features was improved (Morgan and Meyer, 2005; Schotter et al., 2013; Ganmor et al., 2015; Wijdenes et al., 2015; Castelhano and Pereira, 2018; Stewart and Schütz, 2018; Huber-Huber et al., 2019; Buonocore et al., 2020; Kong et al., 2021). Electrophysiological studies have found that brain responses elicited by visual objects was faster (Edwards et al., 2018; Huber-Huber et al., 2019) and had a reduced amplitude (Ehinger et al., 2015; De Lissa et al., 2019; Huber-Huber et al., 2019; Buonocore et al., 2020) when it was previewed. These studies provide evidence for a preview benefit using paradigms in which saccades were controlled. We here complement these findings by investigating the time course of parafoveal and foveal processing during free visual exploration.

The third function of parafoveal previewing is to support trans-saccadic memory (Melcher and Colby, 2008; Cavanagh et al., 2010; Herwig, 2015). As a visual scene is perceived as stable despite eye movements, information preceding each saccade must be integrated with visual input following the saccade. Eye-tracking studies found that the parafoveal and the post-saccadic foveal fixation were combined using a weighted sum (Ganmor et al., 2015; Wolf and Schütz, 2015). Another study found trans-saccadic perceptual fusion of two different stimuli (Paeye et al., 2017). Electrophysiological studies showed that low- and high-level features of presaccadic objects could be classified ∼100–200 ms after the saccade (Edwards et al., 2018; Fabius et al., 2020). This information disappeared when no saccade was performed (Edwards et al., 2018). There is both behavioral and electrophysiological evidence supporting trans-saccadic visual memory from studies constraining saccades. The third aim of our study was to complement these findings by testing whether presaccadic visual features were reflected in the brain data after saccades in free visual exploration.

Here, we investigate the neuronal dynamics associated with feature- and category-related processing of parafoveal objects during free visual exploration using eye-tracking and magnetoencephalography (MEG) recordings. Our main finding was that the feature and category of foveal images and parafoveal saccade goals can be identified in succession from the MEG data within ∼200 ms, providing evidence for fast category parafoveal previewing.

## Materials and Methods

### Participants

Thirty-six participants (29 females, 1 left-handed, mean ± SD age: 21.4 ± 3 years) were included in the study. All participants had a corrected-to-normal vision, were free from medication affecting the central nervous system, and reported no history of psychiatric or neurological disorders. The study was approved by the University of Birmingham Ethics Committee. All participants gave their written informed consent and received a monetary compensation or course credits for their participation.

### Stimuli

**Figure 1.**
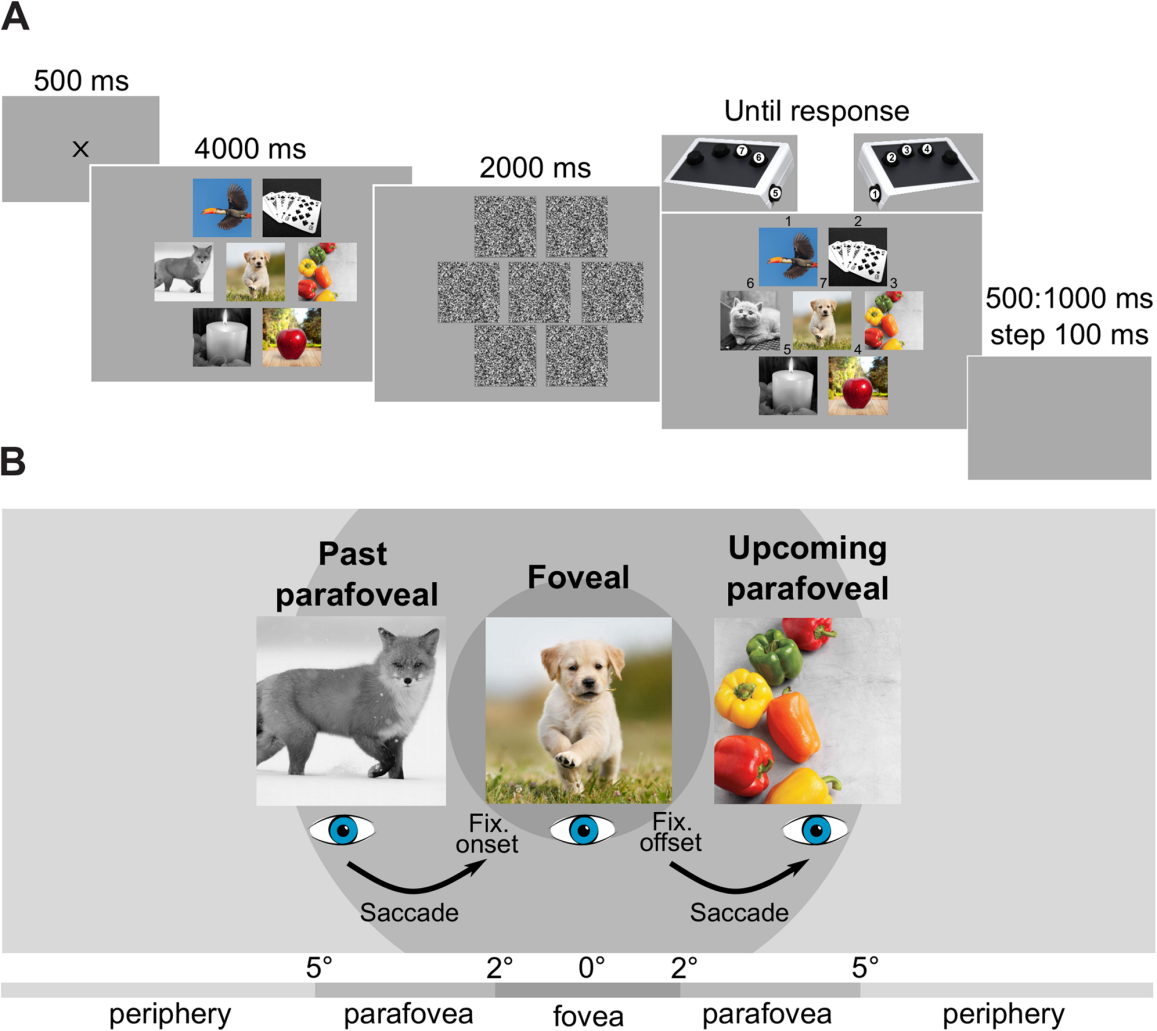
Visual exploration of natural images by saccades. ***A***, Each trial started with the presentation of a central fixation cross for 500 ms. Then followed seven natural images displayed for 4,000 ms. Natural images were shown using either a gray or color scale, and they belong to one of the three different categories: animal, food, or object. The proportion of color and categories were balanced within and between trials. Participants were asked to freely explore the images and eye movements were allowed. Then followed a mask for 2,000 ms, after which the seven images were presented again. One of the images had changed: the different image, belonging to the same category and color scale as the initial one. In this example, the grayscale fox turned into a grayscale cat (6th position). The participants had to identify, without time limit, the image that had changed. A number was presented above each image, as well an imaging showing the link between the image numbers and the response buttons. Each trial was interleaved with a 500–1,000 ms random delay. ***B***, The processing of the color and the category of natural images was investigated in three conditions: (1) The fixated image in the fovea. (2) Upcoming image in the parafovea, corresponding to the image that will be fixated after the saccade. (3) Past image in the parafovea, corresponding to the image that was viewed before the current fixation.

The stimuli used were fixation crosses, natural images, and masks. The fixation cross was black, with arms of 0.2 degrees of visual angles (°) of length and 0.05° of width. In total, 1,500 natural images of our three categories, animal, food, and object, were selected from the THINGS database (Hebart et al., 2019). Visual objects within the same category belong to the same object classes. The natural images were 3° × 3° presented in color or gray scale. In each trial, one image was displayed in the center of the screen, surrounded by six other images (at 0, 60, 120, 180, 240, and 300°), with a 1° distance between each other's borders (Fig. 1*A*). The proportion of image categories and gray versus color scales was balanced within and between trials. The 3° × 3° masks were patches of random gray pixels.

### Procedure

Participants were seated comfortably in the MEG gantry, 145 cm from the projection screen in a dimly lit magnetically shielded room. Participants performed 10 blocks of 30 trials, with the presentation of seven images per trial. In total, 2,100 images were presented throughout the experiment, with ∼20% of repeated images. As shown in Figure 1*A*, each trial began when participants were successfully maintaining fixation, with the presentation of a fixation cross for 500 ms, presented on a gray background. Then seven natural images were presented for 4,000 ms. Participants were instructed to freely explore the seven images and saccades were allowed. A mask was then presented for 2,000 ms after which the seven images were presented again, except for one image that was changed (a different image, belonging to the same category and color scale as the initial one, e.g., a grayscale fox turning into a grayscale cat; Fig. 1*A*). Participants had to identify which image was different from the initial presentation. They had no time limit to respond. A number (1–7) was presented above each image, as well as a figure showing the link between the image numbers and the response buttons. The trials were separated by random intervals varying from 500 to 1,000 ms (in 100 ms steps).

### Equipment

The experimental protocol was designed using the Psychtoolbox 3.0.12, implemented in Matlab 2015b (The MathWorks). Visual stimuli were displayed with a PROPixx Projector (VPixx Technologies), on a 71.5 by 40.2 cm projection screen (1,920 by 1,080 pixels; 120 Hz refresh rate).

#### MEG

MEG data were acquired using a 306-sensor TRIUX Elekta Neuromag system with 204 orthogonal planar gradiometers and 102 magnetometers (Elekta). Participants were seated under the MEG gantry with the back rest at 60° angle. The data were bandpass filtered from 0.1 to 330 Hz and then sampled at 1,000 Hz.

Prior to the MEG study, a Polhemus Fastrak electromagnetic digitizer system (Polhemus) was used to digitize the locations of three fiducial points (nasion, left and right peri-auricular points) and of four head position indicator coils (HPI coils). Two HPI coils were placed on the left end right mastoid bone, and the two others on the forehead with at least 3 cm distance. At least 300 extra points on the scalp were additionally digitalized.

#### Eye tracker

An infrared video-camera system (EyeLink 1000 Plus, SR Research) was used to record participants’ eye movements sampled at 1,000 Hz. A 9-point calibration was performed at the beginning of the experiment and one-point drift corrections were applied at the beginning of every block. The defaults parameters from EyeLink 1000 Plus system were used (EyeLink 1000 User Manual) to identify the following eye metrics: Fixations, Saccades, and Blinks. Saccades were detected by the velocity (threshold of 30°/s) and the acceleration (threshold of 8,000°/s^2^) of eye movements. Blinks were identified when the pupil size was very small or when the pupil in the camera was missing or severely distorted by eyelid occlusion. Fixations corresponded to the absence of Saccades or Blinks events.

### Analyses

Analyses were performed with custom software written in Python 3.10.9 (Python Software Foundation. Python Language Reference. Available at http://www.python.org) and figures were plotted with *Matplotlib* library (Hunter, 2007). MEG analyses were performed using MNE 1.0.3 (Gramfort et al., 2013).

### Behavioral analysis

Behavioral performance was computed as percentage of correct responses, and mean and median reaction times for correct and incorrect trials, for each participant.

### Eye data analysis

The following eye metrics were extracted from the EDF files provided by the EyeLink toolbox, during the initial presentation of the images: number of fixations per trial, fixation durations, number of saccades per trial, and saccade duration, for correct and incorrect trials. We further derived the fixation metrics (number of fixations per trial and fixation durations) for the target image for correct and incorrect trials, in order to specifically investigate whether the behavioral performance could be predicted by the fixation metrics on the target image.

### MEG analysis

#### Preprocessing

MEG data were preprocessed following the standards defined in the FLUX Pipeline (Ferrante et al., 2022). Continuous head movements were estimated by computing the time-varying amplitude of the HPI coils. Sensors with no signal or excessive artifacts were removed using the *mne.preprocessing.find_bad_channels_maxwell* function, and a low-pass filter at 150 Hz was applied to remove the activity from the HPI coils. A Maxwell filter including spatiotemporal signal-space separation (tSSS) was applied to the MEG signal. This procedure removes low-frequency artifacts and performs head movement compensation. Muscle artifacts, defined as activity in the 110–140 Hz band exceeding a *z*-score threshold at 10, were annotated as artifactual. Trials with muscle artifacts were further rejected. An ICA decomposition was performed on the data bandpass filtered at 1–40 Hz. The components were inspected visually for each participant to remove the ocular and cardiac artifacts’ activity in the unfiltered MEG data (typically 3–5 components in each participant).

#### Epoching

Data were extracted in −1 to +1 s epochs aligned to the fixation onset on the images displayed during the initial presentation (fixations on the background were not analyzed). Epochs with a sensor activity exceeding a 5,000 fT/cm threshold for the gradiometers, and 5,000 fT threshold for the magnetometers, were rejected. Epochs were then downsampled to 500 Hz. We discarded epochs with fixation durations below 80 ms and above 1,000 ms. As explained in Figure 1*B*, the epochs were then labeled according to the color (color vs. gray) and the category (animal, food, or object) of the following:

1. Parafoveal past images (fixated before the current image)
2. Foveal images (currently fixated image)
3. Parafoveal upcoming images (fixated after the current image)
4. One parafoveal remaining image (not fixated either before either after the current image)

The parafoveal visual field encompasses objects between 2 and 5° of eccentricity. Consequently, past images viewed before the current fixation in the periphery were not analyzed. Similarly, upcoming images in the periphery relative to the current fixation were not analyzed. Remaining images in the periphery relative to the current fixation were also not analyzed (Fig. 1*B*).

In addition, we did not consider parafoveal past images already visited during the trial, as well as for parafoveal upcoming images, and foveal images. Eventually, upcoming, past, and remaining parafoveal images with features that matched the foveal images’ features were discarded. For example, if the foveal image was a grayscale fox, we did not consider parafoveal images that were gray scale or depicted an animal. This limits the possibility that classification of the parafoveal images was influenced by processing of the foveal image.

#### Multivariate pattern analysis

Multivariate pattern analysis (MVPA) was applied to the MEG data to investigate whether the brain pattern associated with color categorization (gray vs. color) and object categorization (animal vs. food vs. object) can be classified in relation to parafoveal past images, foveal images, and parafoveal upcoming images (Fig. 1*B*). For classification, we used a linear support vector machine (Cortes and Vapnik, 1995) from *Scikit-learn* library (Pedregosa et al., 2011), with a 10-fold cross-validation procedure. Epochs were cropped from −0.5 to +0.5 s including electrophysiological activity from both the gradiometers and the magnetometers (306 sensors). For each time point, we considered the data in a 50 ms time window (25 samples centered around the time point), resulting in a feature vector with 25× N-sensor samples (also termed time-delay embedding; code available at Rohrbacker, 2009). It permits a greater resilience to the varying activation delays across participants. To further increase the signal-to-noise ratio, we averaged 10 trials (randomly selected) in the training set and in the testing set to create the so-called super-trials for each category (Isik et al., 2014; Grootswagers et al., 2017). The creation of super-trials and the classification were repeated 10 times, and the final classification performance was obtained by averaging the classification rates across the 10 repetitions. The classification rate was reported as area under the curve (AUC).

The MVPA was applied to the parafoveal past images, the foveal images, the parafoveal upcoming images, and the parafoveal remaining images. We investigated whether the classifier could disentangle grayscale versus color images and the category. Note that for the category, we averaged the performance over the three classifications: animal versus food, animal versus object, and food versus object. The MVPA was computed for each participant, and the classification performance were averaged across participants.

In addition, a MVPA with generalization across time was performed (King and Dehaene, 2014) on past parafoveal, foveal, and upcoming parafoveal image. The classifier was trained at a given time point and tested on every time points, resulting in a 2D matrix of classification rate, with the diagonal corresponding to when the trained time point matches the testing time point (i.e., MVPA across time, trained and tested on the same time points).

To investigate whether the classification performance was modulated by the behavioral performance, the MVPA was further applied to the foveal images and the parafoveal upcoming images, separately for correct and incorrect trials, for the color and the category. The decoding performance reflects how well the brain patterns associated with different conditions can be distinguished. Therefore, we hypothesized that correct trials, which require the identification of the color and the category of the images, would be associated with higher decoding accuracy. A subsampling procedure was added to the super-trials generation to have an equal number of correct and incorrect trials per participant. In addition, the MVPA was applied to each color and category conditions for correct versus incorrect trials. This analysis would allow to provide direct evidence for a link between behavioral and classification performance.

### Experimental design and statistical analysis

The experiment was performed as a within-subjects design; each participant (*n* = 36) completed all conditions.

Behavioral variables were percentage of correct responses and mean and median reaction times. Eye data variables were number of fixations per trial, fixation durations, number of saccades per trial, and saccade durations. The within-subject factor was participants’ response. To test for significant differences between correct and incorrect trials, two-tailed paired *t* tests from the *Pingouin* library (Vallat, 2018) were performed.

For electrophysiological analysis, MVPA was conducted at the feature (gray scale vs. color scale) and the category (animal vs. food vs. object) levels, on four conditions, described in detail above (see above, Epoching): parafoveal past images, foveal images, parafoveal upcoming images, and one parafoveal remaining images (not fixated either before either after the current image). MVPA was further computed on correct versus incorrect trials for two conditions. The main variables were classification performance, reported as AUC, and the latency of the classification peaks.

To investigate whether the classification performance was above the chance level, we used a cluster permutation approach to control for multiple comparisons over time points (Nichols and Holmes, 2002; Maris, 2012; Winkler et al., 2014). For each of the 1,500 repetitions, we subtracted the chance level (0.5) to the classification performance, randomly multiplied them by 1 or −1 with equivalence across participants, and computed an independent *t* test against zero using the *Scipy* library (Virtanen et al., 2020), at each time point. The maximum *t* value was considered at each repetition, leading to a distribution of *t* values, from which we extracted a threshold *t* value (alpha = 5%). The consecutive *t* values above the threshold *t* value formed significant temporal cluster. The time window associated with the temporal cluster was reported for description purposes. The same cluster permutation approach was used to compare the classification performance between correct and incorrect trials, with a random assignment of the correct and incorrect conditions across participants for each of the 1,500 repetitions, and independent *t* tests conducted at each time point.

To evaluate the latency of the classification peaks, we identified the time points of peaking in each participant in the intervals where decoding performance were above chance level, for both the foveal and parafoveal images, according to fixation onset for the different conditions (color, 60–235 ms interval; category, 160–200 ms interval). The pairwise difference between foveal and parafoveal latencies were computed for each participant and compared against a null difference with two-tailed paired *t* test against 0 (*Pingouin* library). Log10 Bayes factor (BF10) were also computed to test evidence for and against the null hypothesis. The peak latencies for foveal and upcoming parafoveal images were also directly compared with two-tailed paired *t* test.

Although the 50 ms sliding time window induced temporal smoothing in the classification performance, the smoothing was identical between conditions, and it did not affect the timing of the peak. As a result, while comparison between foveal and parafoveal conditions of the classification onset will be impacted by the temporal smoothing, comparison of classification peaks is valid.

### Data and code accessibility

Behavioral, eye, and MEG data are available on a Birmingham University server, and codes to perform the main analyses are available at https://github.com/CamilleFakche.

## Results

Participants were asked to freely explore seven natural images using eye movements in 4 s long trials (Fig. 1*A*). The images could be in color or gray scale and belong to one of the three categories: animal, food, or object. These images were selected from the THINGS database (Hebart et al., 2019). The display was then masked for 2 s, and then six of the images were presented again while one was changed (a different image from the same category and color scale). The task of the participants was to identify the changed image by button press. The aim of the task was to encourage participants to explore the seven images during the initial presentation.

### Behavioral and eye data

**Figure 2.**
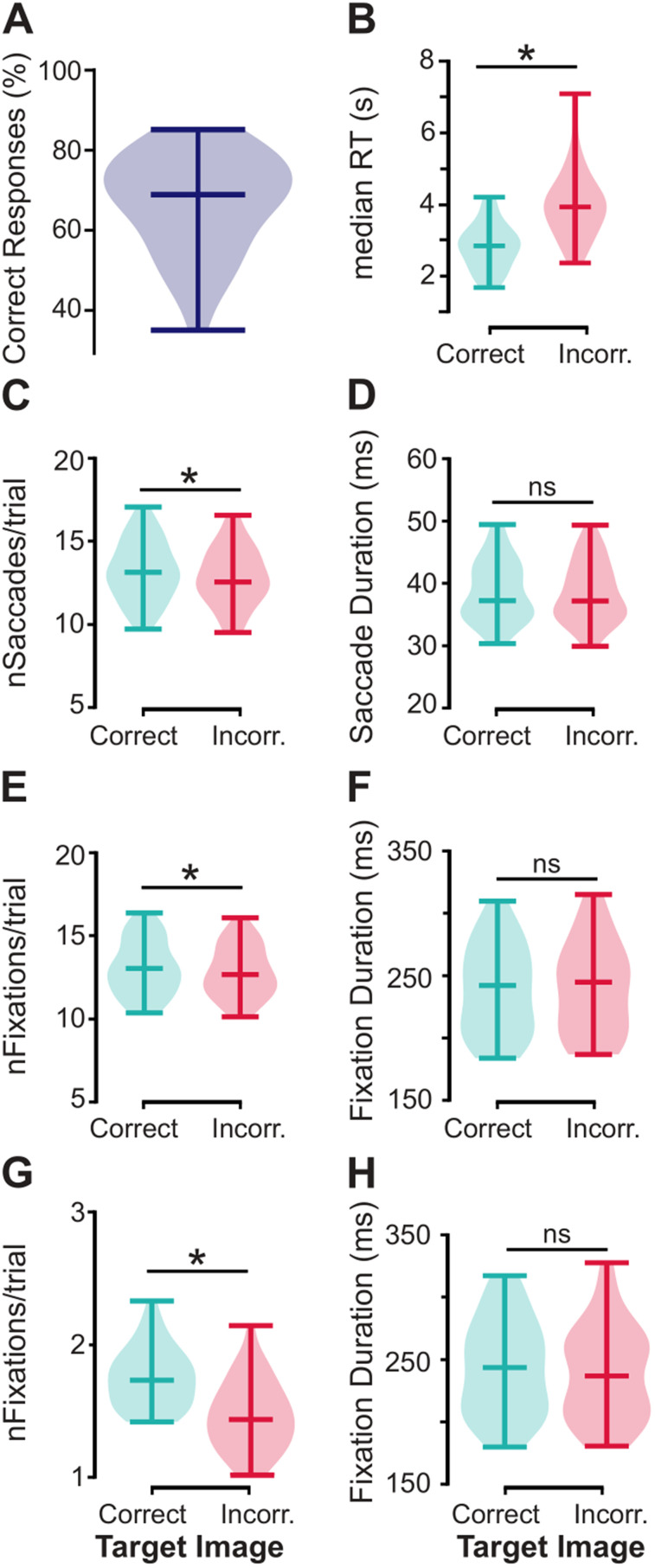
The behavioral performance was predicted by how often the images were visited. ***A***, Percentage of correct responses regarding identifying the image that had changed, i.e., target image, ***B***, The median reaction times (RT) were significantly longer for incorrect compared with correct trials (two-tailed paired *t* test: *t*_(35)_ = −11.4, *p* < 0.001, Cohen's *d* = 1.43, CI95% = [−1.41, −0.98]). ***C***, The number of saccades per trial was significantly higher for correct compared with incorrect trials (two-tailed paired *t* test: *t*_(35)_ = 6.8, *p* < 0.001, Cohen's *d* = 0.21, CI95% = [0.26, 0.49]). ***D***, The mean saccade durations (i.e., eyes in flight) did not differ between correct and incorrect trials. ***E***, The number of fixations per trial was significantly higher for correct compared with incorrect trials (two-tailed paired *t* test: *t*_(35)_ = 5.2, *p* < 0.001, Cohen's *d* = 0.16, CI95% = [0.15, 0.35]). ***F***, The average fixation durations on each object were similar between correct and incorrect trials. The fixation metrics were further computed for the target images specifically. ***G***, The number of fixations per trial for the target image was significantly higher for correct compared with incorrect trials (two-tailed paired *t* test: *t*_(35)_ = 8.3, *p* < 0.001, Cohen's *d* = 1.16, CI95% = [0.22, 0.36]). ***F***, No significant difference between correct and incorrect trials for the fixation durations on target images. The horizontal bar in the violin plots indicates the median value in ***B***, and the mean value in (***A***), ***C–F***. Dark blue plot, all trials. Cyan plots, correct trials. Pink plots, incorrect trials. ns, nonsignificant. *p < 0.001.

All participants (*n* = 36) were able to identify the changed image as reflected by a percentage of correct responses being all above chance (66.8 ± 12.3%; mean ± SD; Fig. 2*A*). As expected, the median reaction time was longer for incorrect (4,010 ± 973 ms) compared with correct (2,813 ± 640 ms) responses (two-tailed paired *t* test: *t*_(35)_ = −11.4, *p* < 0.001, Cohen's *d* = 1.43, CI95% = [−1.41, −0.98]; Fig. 2*B*). Similar results were observed for mean reaction times, with longer response times for incorrect (4,242 ± 1,038 ms) than for correct trials (3,183 ± 639 ms; two-tailed paired *t* test: *t*_(35)_ = −10.8, *p* < 0.001, Cohen's *d* = 1.21, CI95% = [−1.26, −0.86]).

For the eye movement metrics, the number of saccades per trial was significantly higher for correct (13.2 ± 1.8; mean ± SD) compared with incorrect trials (12.9 ± 1.7; two-tailed paired *t* test: *t*_(35)_ = 6.8, *p* < 0.001, Cohen's *d* = 0.21, CI95% = [0.26, 0.49], Fig. 2*C*). There was no significant difference between correct and incorrect trials in regard to the saccade durations (two-tailed paired *t* test: *t*_(35)_ = 1.4, *p* = 0.16, Cohen's *d* = 0.04, CI95% = [−0.09, 0.5]; Fig. 2*D*). Participants did on average 3.26 ± 0.44 saccades per second, in line with the previous literature (Skaramagkas et al., 2021). Similarly, the number of fixations per trial was significantly higher for correct (13.1 ± 1.6) than that for incorrect trials (12.8 ± 1.5; two-tailed paired *t* test: *t*_(35)_ = 5.2, *p* < 0.001, Cohen's *d* = 0.16, CI95% = [0.15, 0.35]; Fig. 2*E*), but no difference was observed for the fixation durations (two-tailed paired *t* test: *t*_(35)_ = −0.8, *p* = 0.4, Cohen's *d* = 0.03, CI95% = [−3.31, 1.5]; Fig. 2*F*). We further computed the fixation metrics for the target images (that changed after the 2 s masking). Participants fixated significantly more often on the target images of correct (1.8 ± 0.2 fixations per trial) compared with incorrect trials (1.5 ± 0.3; two-tailed paired *t* test: *t*_(35)_ = 8.3, *p* < 0.001, Cohen's *d* = 1.16, CI95% = [0.22, 0.36]; Fig. 2*G*). The fixation durations, however, were similar (two-tailed paired *t* test: *t*_(35)_ = 1.6, *p* = 0.11, Cohen's *d* = 0.12, CI95% = [−1.15, 10.36]; Fig. 2*H*). The mean fixation duration on each of the natural images was 240 ± 36 ms. In summary, performance could be predicted by how often the participants visited the target image, as well as all images.

### MEG data

MVPA was applied to the MEG data aligned according to the fixation onset on foveal images to investigate whether we can classify the color (gray scale vs. color scale) and the category (animal vs. food vs. object) of the following, as illustrated in Figure 1*B*:

1. Foveal images (currently fixated images)
2. Parafoveal upcoming images (viewed after the current image)
3. Parafoveal past images (viewed before the fixation on the current image)
4. One parafoveal remaining image (not viewed after, not viewed before the current image)

### Foveal decoding of features and categories

**Figure 3.**
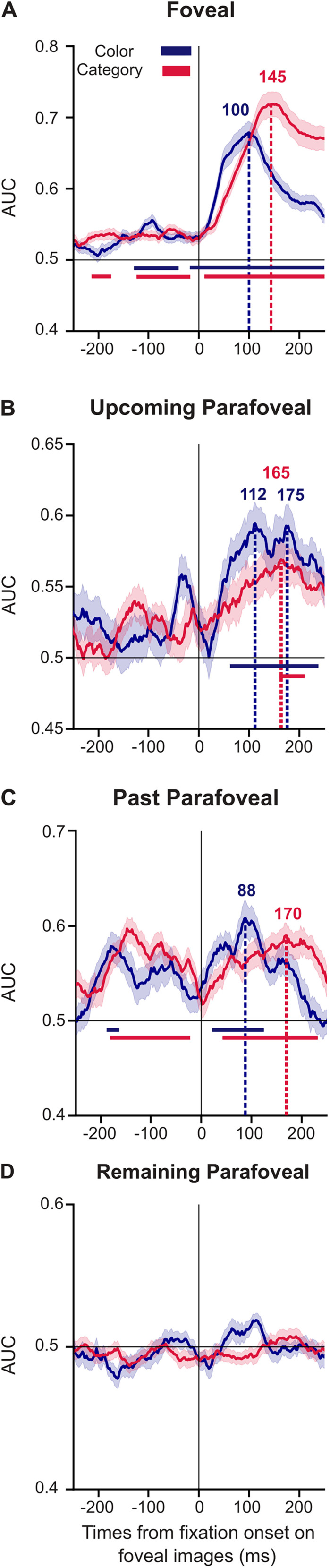
Decoding foveal, upcoming parafoveal, and past parafoveal images at the feature and category level. The feature and the category of foveal, upcoming parafoveal, and past parafoveal images can be identified from the MEG data during the foveation as shown by the classification analysis across time. The MEG data was aligned to fixation onset on foveal images (10-fold cross-validation; [−250; +250] ms; area under the curve, AUC). Classification for color (grayscale vs. color: blue line), and category (animal vs. food vs. object: red line), for ***A***, foveal images (currently fixated images), ***B***, upcoming parafoveal images (viewed after the current image), ***C***, past parafoveal images (viewed before the fixation on the current image). ***D***, remaining parafoveal image (not viewed after, not viewed before the current image). The shaded areas reflect the standard error of the mean (SEM). A cluster permutation approach was used to identify significant temporal cluster (*p* < 0.01). The color horizontal lines described the time window associated with the temporal clusters.

For foveal fixations, the classifier was trained and tested at each time point (using a 50 ms sliding time window and 10-fold cross-validation). To reduce contamination by the upcoming or previous saccades, we focused our interpretation of the classifier results in the −250 to 250 ms interval aligned to the fixation onset. As seen in Figure 3*A* (blue curve), the classifier could reliably distinguish the color of the foveal images well above chance level (AUC of 0.5) in the −130 to −40 ms interval and the −15 to 250 ms interval (*p* < 0.01; cluster permutation approach controlling for multiple comparisons over time; Nichols and Holmes, 2002; Maris, 2012; Winkler et al., 2014). Classification performance (AUC) gradually built up until peaking to 0.68 at 100 ms, after which it decreased. The classifier could also reliably distinguish the brain patterns associated with the category of the foveated images. The performance of the classifier was above chance in the −210 to −175 ms interval, the −125 to −20 ms interval, and the 10–250 ms interval (*p* < 0.01, cluster permutations approach). The category classification gradually built up until peaking to 0.72 at 145 ms, after which it decreased (Fig. 3*A*, red curve). These results demonstrate that foveal images are processed in succession at the feature and category level during free visual exploration. Note that the classification performance for both the color and the category began before the fixation onset on the foveal image (color, −130 to −40 ms; category, −210 to −175 ms, −125 to −20 ms), providing evidence of a parafoveal previewing; however, the classification accuracy increased dramatically ∼60 and ∼130 ms after fixation for respectively feature and category decoding.

### Decoding of parafoveal upcoming images at the feature and category level

Next, we investigated the parafoveal previewing at the feature and category level. We trained and tested the same classifier at each time point for the data aligned to the fixation onset on the foveal images, to test whether we can classify the color and category of the upcoming parafoveal images. As shown in Figure 3*B* (blue curve), the classifier could distinguish the color of the parafoveal images, well above chance in the 60–235 ms interval (*p* < 0.01, cluster permutations approach). The decoding peaked to 0.59 at 112 and 175 ms. Similarly, the classifier was able to differentiate the category of the parafoveal images. The performance was above chance in the 160–200 ms interval (*p* < 0.01, cluster permutations approach), with a peak at 0.57 at 165 ms (Fig. 3*B*, red curve). We here also observed a gradual increase and decrease of the performance around the peak, for both the color and feature decoding.

In theory, the classification performance for the upcoming parafoveal images after *t* = 0 s should be identical to the classification performance for the foveal images before *t* = 0 s. However, this was not the case for two reasons. First, the electrophysiological data were epoched according to fixation onset. While the data are perfectly aligned after *t* = 0 s, the preceding variability in the intersaccadic interval introduced noise to the interval before *t* = 0 s. Second, the epoch selection of upcoming parafoveal images involved fewer trials compared with the selection of foveal images, creating a difference in signal-to-noise ratio across conditions.

Although the classification performance did not reach significance before *t* = 0 s, it seemed that some information related to the feature and category of upcoming parafoveal images started to emerge in the brain data. This result suggested that participants may plan multiple saccades while initiating and executing only one saccade.

In summary, our analysis shows that during unconstrained visual exploration, there is parafoveal previewing of upcoming saccade targets at both the feature and the category levels.

### Decoding of parafoveal past images at the feature and the category level

We then tested whether the feature and category of the image previously seen could be decoded. The classifier was trained and tested on data aligned according to fixation onset on the foveal images, to test whether we could classify the color and the category of the foveal images viewed just before the saccade. As seen in Figure 3*C* (blue curve), we found that the classifier was able to categorize the color of the parafoveal past images in the −185 to −165 ms interval (*p* < 0.01, cluster permutations approach). This is unsurprising as this is likely to reflect the image being on the fovea at *t* < 0 ms. More interestingly, we found robust decoding of the past parafoveal image color in the 20–120 ms interval following fixation. The classification peaked to 0.61 at 88 ms. Similarly, the classifier could also identify the category of the past parafoveal images in the −175 to −25 ms interval and the 45–235 ms interval (*p* < 0.01, cluster permutations approach; Fig. 3*C*, red curve), with a peak to 0.59 at 170 ms. These results demonstrate that the neuronal activity reflecting both the feature and the category information of a given image is sustained after the subsequent saccade.

### Absence of decoding accuracy for parafoveal non-targets

We additionally computed classification accuracy for parafoveal images that were not targets of upcoming saccades, nor targets of previous saccades. The classifier was trained and tested on data aligned according to fixation onset on the foveal images. As seen in Figure 3*D*, the classifier was not able to distinguish the color (blue curve) and the category (red curve) of parafoveal images (AUC at the chance level). These results demonstrate that parafoveal images can only be decoded if they are included in the saccade goal, a notion consistent with presaccadic attention.

### Temporal generalization

**Figure 4.**
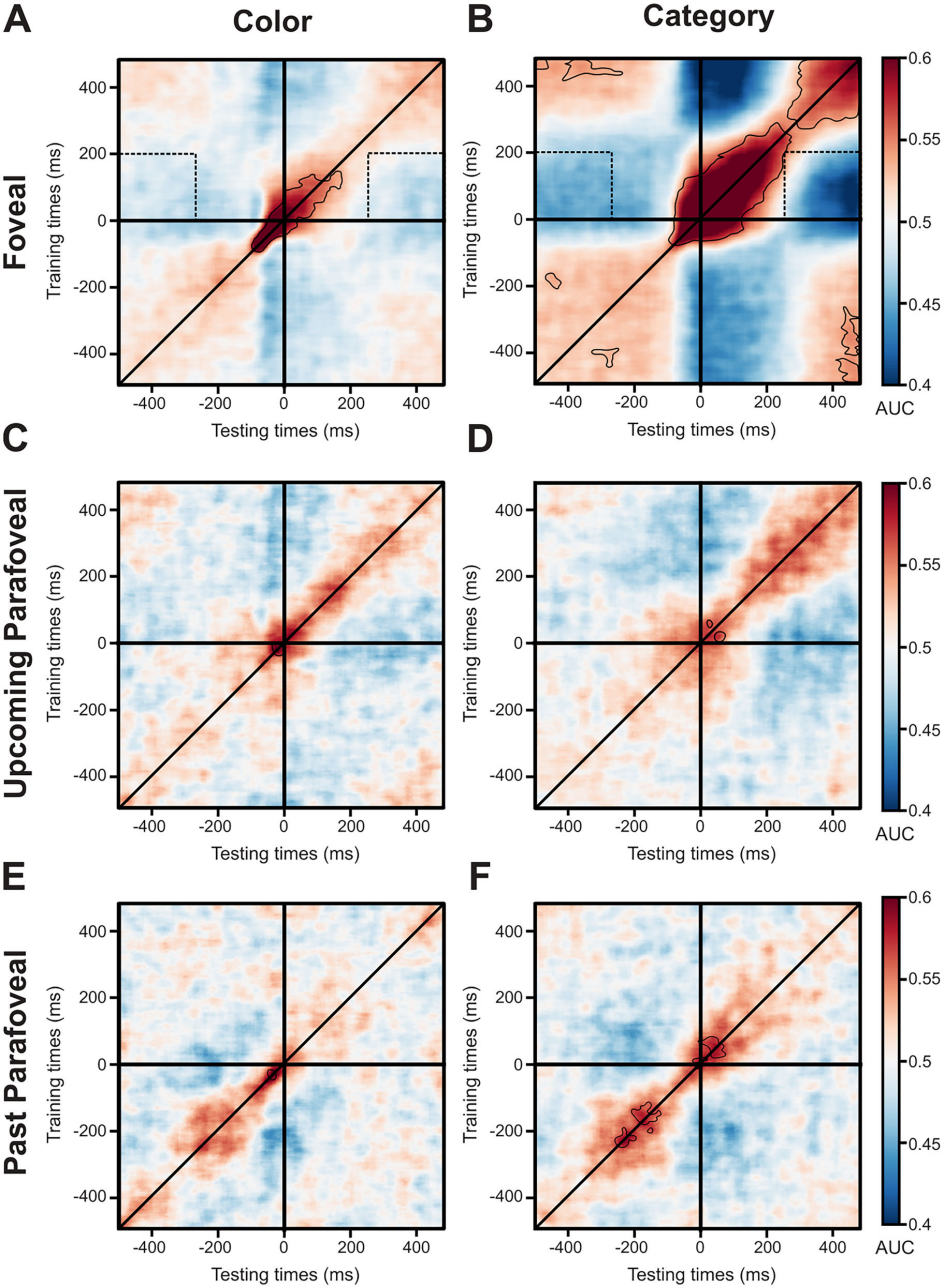
The decoding for color classification was transient while stable across an ∼200 ms interval for category classification. Temporal generalization analysis on MEG data aligned to fixation onset on the foveal images ***A***, color classification, foveal images; ***B***, category classification, foveal images; ***C***, color classification, upcoming parafoveal images; ***D***, category classification, upcoming parafoveal images; ***E***, color classification, past parafoveal images; ***F***, category classification, past parafoveal images. The color scale reflects the classification performance (area under the curve, AUC). The solid black outlines identify the significant time points with correction for multiple comparisons (*p* < 0.05, cluster permutations approach). The dotted line boxes correspond to the time window where brain data are trained on foveal images and tested on upcoming parafoveal and past parafoveal images. A succinct diagonal pattern was observed in the temporal generalization of color classification, while a square generalization matrix was noticed for the category classification. These results suggest that color processing was transient, while category processing was stable for ∼200 across time.

To quantify the temporal generalization, the classifier was trained on all time points and tested on all time points, for color and category classification. The temporal generalization analysis results in 2D matrices of classification performance, with the diagonal corresponding to when the classifier was trained and tested at the same time points (Fig. 3). The classification rate above chance at the off-diagonal reflects that the training for these time points enables the classifier to generalize to other time points (Fig. 4). This approach allows us to investigate how stable the neural code is across time (King and Dehaene, 2014). For the color classification, a succinct diagonal pattern was observed in the foveal condition (Fig. 4*A*; *p* < 0.05, cluster permutations approach). These results suggest that the color of natural images was processed transiently by the brain. For the classification of the images’ category, a square-like generalization matrix was observed off the diagonal in the foveal condition (Fig. 4*B*; *p* < 0.05, cluster permutations approach). This pattern suggests that the images’ category was encoded by a stable ensemble of neurons across time over a couple of hundred milliseconds. In the upcoming parafoveal (Fig. 4*C*,*D*) and the past parafoveal (Fig. 4*E*,*F*) conditions, the classification of color and category showed only short windows of significant effects along the diagonal (*p* < 0.05; cluster permutations approach). Note that the discrepancy between the significant time windows observed during foveation for the classification across times and the temporal generalization, for the upcoming parafoveal (Fig. 3*B*, color, 60–235 ms; category, 160–200 ms; Fig. 4*C*,*D*, short significant time window, few milliseconds, around fixation onset) and the past parafoveal images (Fig. 3*C*, color, 54–62 ms, 84–122 ms; category, 0–235 ms; Fig. 4*E*,*F*, short significant time window <50 ms) probably came from the increase of multiple comparisons in the temporal generalization, leading to a more restrictive *p* value threshold. In addition, the temporal generalization allowed us to investigate whether the brain patterns associated with feature and category classification were shared between foveal, upcoming parafoveal, and past parafoveal conditions. Indeed, if the brain patterns were identical, we should observe above chance classification performance when training on the time points corresponding to the foveal condition and testing on the time window associated with upcoming and past parafoveal conditions. However, the performance was not above chance level in these segments (Fig. 4*A*,*B*, dotted line boxes). In sum, although the neuronal pattern that encodes the color of natural images was transient, the pattern encoding the category was stable across time. The temporal generalization suggests that brain patterns encoding the color and the category differ between foveal, upcoming parafoveal, and past parafoveal images.

### Not purely parallel processing of foveal and parafoveal color information

**Figure 5.**
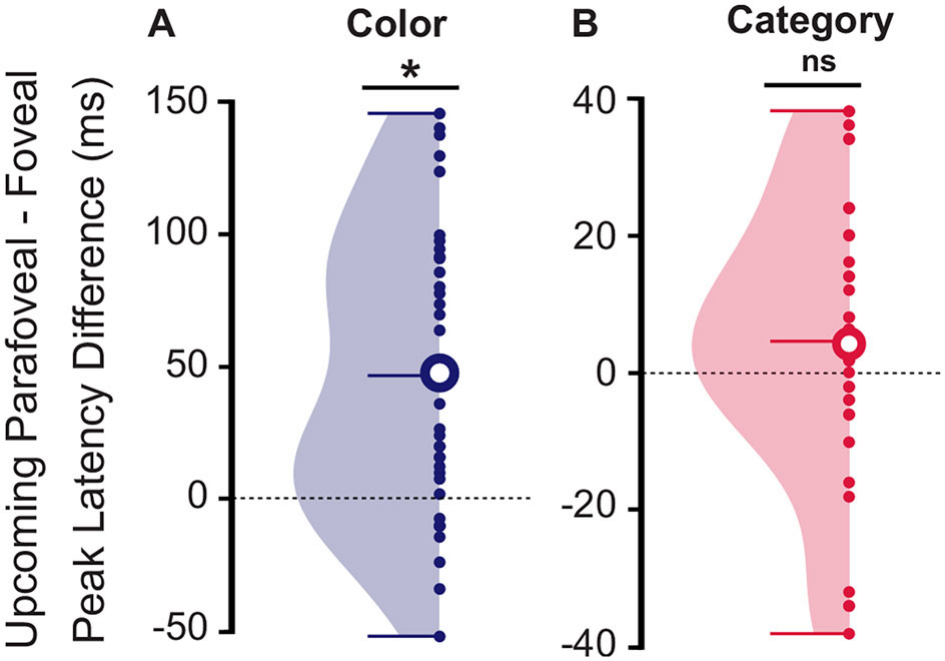
Color decoding peaked later for upcoming parafoveal compared with foveal images. The peak latency differences between foveal and upcoming parafoveal images computed during the foveation for the *n* = 36 participants. ***A***, Color classification. The peak latency difference was significantly above 0 (two-tailed paired *t* test: *t*_(35)_ = 5.12, *p* < 0.001, Cohen's *d* = 1.21, CI95% = [0.03, 0.07], BF10 > 100). The peak of classification for parafoveal images was ∼45 ms later than for foveal images. ***B***, Category classification. The peak latency difference was not significantly different from 0 (two-tailed paired *t* test: *t*_(35)_ = 1.33, *p* = 0.19, Cohen's *d* = 0.31, CI95% = [−0, 0.01], BF10 = 0.4). There was no strong evidence in favor or against the null hypothesis. The horizontal bar in the violin plots indicates the mean value. Filled circle, participant's difference. Empty circle, average difference across *n* = 36 participants.

Whether the processing of the foveal and parafoveal information is purely parallel or in accordance with the pipelining theory remains to be determined (Jensen et al., 2021). To address this question, we extracted the latency of the peaks of classification for each participant during the foveation, for the foveal and upcoming parafoveal images, with respect to color and category classification intervals above chance level (Fig. 3*A*,*B*), and computed the pairwise difference between foveal and upcoming parafoveal latencies for each participant. For the color classification, the peak latency differences between foveal and upcoming parafoveal images were significantly different from the null hypothesis (two-tailed paired *t* test: *t*_(35)_ = 5.12, *p* < 0.001, Cohen's *d* = 1.21, CI95% = [0.03, 0.07]). The mean peak latency difference was 47 ± 54 ms (Fig. 5*A*), and the Log10 Bayes factor was >100 (BF10 = 1,863), providing decisive evidence in favor of the alternative hypothesis, i.e., the peak latency difference between foveal and parafoveal images was non-null [Van der Linden et al. (2018), their Table 3]. The direct comparison between the foveal (108 ± 39 ms) and upcoming parafoveal (155 ± 52 ms) peak latencies confirmed these results (two-tailed paired *t* test: *t*_(35)_ = −5.12, *p* < 0.001, Cohen's *d* = 1, CI95% = [−0.07, −0.03], BF10 = 1,863). For the category classification, we did not find a significant difference between foveal and upcoming parafoveal latency differences (two-tailed paired *t* test: *t*_(35)_ = 1.33, *p* = 0.19, Cohen's *d* = 0.31, CI95% = [−0, 0.01]). The mean peak latency difference was 5 ± 20 ms (Fig. 5*B*). The Log10 Bayes factor was in the 0.33–1 interval (BF10 = 0.4), suggesting anecdotal evidence for the null hypothesis, i.e., the peak latency difference between foveal and parafoveal images was null [Van der Linden et al. (2018), their Table 3]. The direct comparison between foveal (171 ± 14 ms) and upcoming parafoveal (175 ± 15 ms) peak latencies confirmed the results (two-tailed paired *t* test: *t*_(35)_ = −1.33, *p* = 0.19, Cohen's *d* = 0.31, CI95% = [−0.01, 0], BF10 = 0.4). These findings provided evidence that the processing of foveal and parafoveal images at the color level were not purely parallel but associated with a time difference of ∼45 ms between the foveal and the parafoveal images. At the category level, we observed a nonsignificant 5 ms time delay between foveal and parafoveal processing. The Bayes factor did not provide evidence either for null or a non-null time delay between foveal and parafoveal images. Consequently, we cannot conclude whether a parallel or a nonparallel processing occurred at the category level. In sum, our results are more closely in line with pipelining processing than strict parallel processing.

### The decoding of features and categories of foveal images tracks behavioral performance

**Figure 6.**
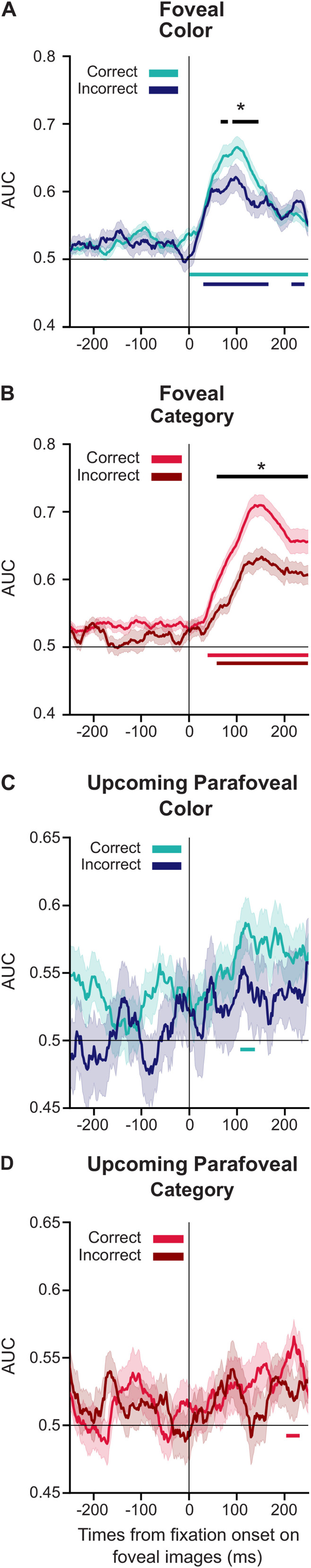
Feature and category decoding of foveal images was higher for correct compared with incorrect trials. ***A***, The feature (gray scale vs. color: blue lines) and ***B***, the category (e.g., animal vs. food: red lines) of foveal images are better identified for correct than incorrect trials in MEG data aligned to fixation onset on foveal images (10-fold cross-validation; [−250; +250] ms; area under the curve, AUC). ***C***, The feature and ***D***, the category of upcoming parafoveal images are only classified for the correct trials. The shaded areas reflect standard error of the mean (SEM). A cluster permutations approach was used to identify the significant temporal cluster. The colored horizontal lines denote the time window associated with the temporal clusters above the chance level (*p* < 0.01), and the black horizontal lines the temporal clusters where correct and incorrect trials were different (*p* < 0.05).

The decoding performance reflects how well the brain patterns associated with different conditions can be distinguished, but do they reflect the perception and maintenance of the objects? We hypothesized that the decoding would predict the behavioral performance. Specifically, we expected to observe higher decoding accuracy for correct trials, considering that the task requires the participants to identify the visual features (color and category) of the natural images. To test this hypothesis, the classifier was trained and tested on correct and incorrect trials separately, on foveal and upcoming parafoveal images. For foveal images, the classifier was able to categorize the color in the 0–250 ms interval and in the 30–165 and 215–240 ms intervals, for correct (light blue curve) and incorrect (dark blue curve) trials respectively (*p* < 0.01, cluster permutations approach; Fig. 6*A*). A significant difference in the classification of features was observed between correct and incorrect trials in the 65–80 and 90–145 ms intervals (we compared correct and incorrect conditions when their performance was above the chance level; *p* < 0.05, cluster permutations approach; Fig. 6*A*). The classifier identified the category of foveal images, in the 35–250 ms interval and in the 55–250 ms interval, for correct (light red curve) and incorrect (dark red curve) trials, respectively (*p* < 0.01, cluster permutations approach; Fig. 6*B*). We found a significant difference in classification performance between correct and incorrect trials in the 55–250 ms interval (correct and incorrect conditions were compared when their performance was above the chance level; *p* < 0.05, cluster permutations approach; Fig. 6*B*). Regarding the upcoming parafoveal images, the classifier was able to identify the color and the category only for correct trials, in the 105–135 ms interval and the 200–230 ms interval, respectively (*p* > 0.05, cluster permutations approach; Fig. 6*C*,*D*). Because the performance for the incorrect trials was not significantly different from the chance level, we did not compare the performance between correct and incorrect trials. We further trained and tested the classifier to distinguish correct versus incorrect trials, for the different images’ color and category, on foveal and upcoming parafoveal conditions. However, the classifier was not able to identify the responses in any conditions (performance was not significantly different from the chance level; *p* > 0.05, cluster permutations approach). Consequently, although the decoding of the foveal images seems to reflect the behavioral performance, we cannot establish direct evidence between the classification and the behavioral performance.

## Discussion

This study aimed to investigate presaccadic attention by uncovering how quickly and to what detail the brain processes visual information when we explore visual images. We applied MVPA to analyze MEG and eye-tracking data of participants viewing sets of natural images belonging to different categories and presented in gray scale or color. The MVPA results showed that the brain processes images both in the fovea and the parafovea at the feature and category levels. The parafoveal feature and category-specific decoding peaked at ∼110 and ∼165 ms, respectively, whereas decoding of fixated objects peaked at ∼100 and ∼145 ms, respectively. The magnitude of the classification of the foveated object also seemed to reflect perceptual performance. Additionally, we found that feature- and category-specific information about previously fixated objects in the parafovea was maintained after the eyes had moved away from them. Interestingly, the feature and category of nontarget parafoveal images could not be decoded, suggesting that visual processing was constrained by the saccade goal. Overall, these findings showed that the brain can simultaneously extract detailed information about both foveal and parafoveal objects within an intersaccadic interval, and this may help to plan the next saccade as well as to construct a representation of the full scene.

We found that the classification of upcoming parafoveal visual objects peaked at the feature and the category level at ∼110 and ∼165 ms, respectively. This is consistent with a previous study using EEG and a paradigm involving controlled eye movements in which they found that the category classification of parafoveal objects peaked at ∼160 ms (Edwards et al., 2018). We complement these findings by identifying a similar time course during unconstrained visual exploration. Another EEG study used free visual exploration and fixation-related potentials to show that the congruency of parafoveal visual target objects with respect to the scene was reflected in the N400 response before saccading to the parafoveal object (Coco et al., 2020). We here extend these findings by demonstrating that the category-specific parafoveal processing builds up and peaks already at ∼165 ms after fixating on the pre-target object. Importantly, the parafoveal processing of the feature and the category was specific to objects of the next saccade goal. Our results provide electrophysiological evidence that category information of the parafoveal object can be extracted sufficiently fast (within ∼200 ms) to impact saccade plans in free visual exploration. Further studies should be done to investigate whether information of the next peripheral object (fixation + 2) can also be extracted during the foveation to impact the second following saccade.

In our study, the classification of feature and category information of foveal objects peaked at 100 and 145 ms, respectively, after the fixation onset. Thus, the parafoveal preview resulted in only a modest if any speed up in the categorization of visual objects after fixations, given that other studies have found a peak in classification at ∼150 ms without previewing (Hung et al., 2005; Cichy et al., 2014; Bezsudnova et al., 2024). These considerations only apply to the peak of classification. The object color and category could be decoded above chance already prior to the saccade, from −130 to −40 ms, and from −15 ms to fixation onset for the color and from −210 to −175 ms, and from −125 to −20 ms for the category (Fig. 3*A*). This provides evidence for feature and category evidence starting to accumulate of parafoveal objects already prior to the saccade. In our design, we did not have a condition preventing parafoveal processing. Other studies using tasks with controlled eye movements (Morgan and Meyer, 2005; Schotter et al., 2013; Ganmor et al., 2015; Wijdenes et al., 2015; Castelhano and Pereira, 2018; Edwards et al., 2018; Stewart and Schütz, 2018; Huber-Huber et al., 2019; Buonocore et al., 2020; Kong et al., 2021) have found evidence for preview benefit, where some suggest that parafoveal previewing reduces the subsequent processing time by at least 30 ms (Pollatsek et al., 1990; Edwards et al., 2018). In future studies, it would be of great interest to extend our design such that previewing is manipulated. This could be done using a gaze-contingent paradigm in which the parafoveal object is replaced (or not) when saccading toward it. Our multivariate approach could then be used to compare the classification time after a preview versus a nonpreview has been performed. This will allow for estimating the precise benefit of parafoveal processing in free visual exploration.

Our study revealed that feature decoding of foveal objects peaked at 100 ms (robust in the −130 to −40 and −15 to 50 ms intervals) and category decoding peaked at 145 ms (robust in the −210 to −175, −125 to −20, and 10–250 ms intervals). The peak decoding of feature and category decoding of upcoming parafoveal objects were identified at ∼110 ms (robust in a 60–235 ms interval) and 165 ms (robust in a 160–200 ms interval), respectively. First, our findings provide evidence against a strict serial mechanism, relying on parafoveal processing starting once the foveal object has been fully identified (Reichle et al., 2009; White et al., 2019). We base this argument on feature processing of the parafoveal visual object (peak at 110 ms, onset at 60 ms) starting already before the category processing of the foveal object (peak at 145 ms, onset before fixation). Although serial preprocessing often has been proposed to explain the brain dynamics in reading, a specific type of visual cognition, recent studies challenged this theory (Pan et al., 2021). In regard to parallel processing (Ludwig et al., 2014; Snell and Grainger, 2019), we did find considerable overlap in the time interval in which foveal and parafoveal objects were decoded both in terms of feature- and category-specific processing. As a challenge to a strict parallel processing scheme, our analysis revealed a significant ∼45 ms delay in the peak decoding between the foveal and parafoveal processing of the color. While there was a difference (∼5 ms) in the peak of category processing, this was not statistically different. Please note that the apparent discrepancy between the peak latency differences between foveal and parafoveal images (participant-by-participant computation; color, ∼45 ms; category, ∼5 ms) and the peak latencies per se (mean across participants computation; color: foveal ∼110 ms, parafoveal ∼100 ms, difference ∼10 ms; category: foveal ∼145 ms, parafoveal ∼165 ms, difference ∼20 ms) came from the inter-subject's variability in the classification performance dynamics. In addition, the color and category processing of parafoveal objects started (onset at 60 and 160 ms, respectively) later than for the foveal object (onset at −15 and 10 ms, respectively). The onsets of feature and category decoding of parafoveal objects were in fact closer in time to the peak of foveal decoding (peak at 100 and 145 ms, respectively). These differences in time suggested that parallel processing cannot fully account for the observed results. These results are closely in line with the idea of a pipelining mechanism (Jensen et al., 2021). According to this idea, several objects can be processed simultaneously but at different levels of the visual hierarchy. The sequential activation of the color-specific processing is aligned with the pipelining mechanism. While we provided evidence against the serial theory, our findings are better aligned with pipelining theory. To further investigate the temporal sequence of foveal and parafoveal processing and saccade execution, trial-by-trial analysis of the timing associated with the visual and oculomotor processing would be highly valuable. Although a leave-one-out procedure may be considered, i.e., computing the foveal and parafoveal feature and category classification performance according to the saccade timing, the computational resources required render this approach highly challenging. Future studies would be necessary to further uncover the temporal dynamics and the associated mechanisms of foveal and parafoveal processing along the visual hierarchy.

We found that the classification performance at the feature and the category level for foveal images tracked the behavioral performance in the memory task (note that we did not find direct evidence between behavioral and classification performance). These findings suggest that the distributed brain responses are related to how well the images are perceived and retained. However, we were not able to link the decoding of parafoveal objects to performance. This may be a matter of low signal-to-ratio as there were not too many incorrect trials. In the future, increasing the number of trials as well as task difficulty would help to determine if the parafoveal processing also reflects memory performance.

Finally, we found that the feature and the category information of past parafoveal images remained present after saccade onset. This finding is in line with studies based on controlled eye movements showing that brain data associated with low- and high-level visual features of past parafoveal objects remained present for 100–200 ms after the saccade (Edwards et al., 2018; Fabius et al., 2020). We here show that these findings generalize to free-viewing conditions. Our finding supports the notion that visual information can be transferred across saccades (Melcher and Colby, 2008; Cavanagh et al., 2010; Herwig, 2015). In future research, it would be interesting to uncover how information from past and current saccades contributes to building the full percept of a visual scene.

The temporal generalization allowed us to investigate whether the distributed brain activity associated with color and category were shared between foveal, upcoming parafoveal and past parafoveal images. Our results suggested that the brain patterns differed between foveal and parafoveal representations of feature and category. This finding is in line with a recent study showing that neural codes for orientation were not shared between foveal and peripheral visual objects within a similar time window (Kandemir and Olivers, 2024). However, they found a generalization of the alpha power-based brain signals in a late time window (∼800–1,200 ms after stimulus onset). A transformation from a local sensory brain pattern to an alpha-based generalized representation would emerge later (Kandemir and Olivers, 2024). Our experimental design makes it impossible to track such a transformation in the current data, but there is clear opportunity in further research to investigate how the foveal and parafoveal brain representations evolve dynamically during free-viewing.

While the participants scanned the display at their own volition, the configuration of the seven images was somewhat artificial. The next step could be to develop the approach to study the exploration of real natural scenes. The objects in the natural scenes as well as their location could be labeled using automatic tools derived from machine learning (Ramík et al., 2014; Wäldchen and Mäder, 2018). If successful, a similar approach could be used for videos. Eventually the study could be conducted outside the laboratory where the brain activity is measured from participants walking in a natural environment with eye-tracking glasses and the brain activity measured using a mobile EEG. In addition, we did not consider the eyes’ trajectories during the saccades to the visual objects. One could imagine that a change in direction between the past, foveal, and upcoming images may alter the efficiency in their processing. Further studies need to be done to investigate the consequence of eye's trajectories on parafoveal processing.

In conclusion, our study provides electrophysiological evidence that parafoveal images are processed at the feature and category levels during free visual exploration, within ∼200 ms after fixation onset. This demonstrates that category information of parafoveal objects can be extracted sufficiently fast to guide saccades during free visual exploration and potentially support the parafoveal preview benefit. Our results also demonstrate that within an intersaccade interval, information about past, current, and upcoming objects is represented in the brain. This information might be part of the building blocks supporting stable perception in the presence of saccades.

## References

[B1] Bezsudnova Y, Quinn AJ, Wynn SC, Jensen O (2024) Spatiotemporal properties of common semantic categories for words and pictures. J Cogn Neurosci 36:1760–1769. 10.1162/jocn_a_02182 38739567 PMC7619145

[B2] Bonitz VS, Gordon RD (2008) Attention to smoking-related and incongruous objects during scene viewing. Acta Psychol 129:255–263. 10.1016/j.actpsy.2008.08.006 18804752 PMC4058766

[B3] Borges MT, Fernandes EG, Coco MI (2020) Age-related differences during visual search: the role of contextual expectations and cognitive control mechanisms. Aging Neuropsychol Cogn 27:489–516. 10.1080/13825585.2019.1632256 31307316

[B4] Buonocore A, Dimigen O, Melcher D (2020) Post-saccadic face processing is modulated by pre-saccadic preview: evidence from fixation-related potentials. J Neurosci 40:2305–2313. 10.1523/JNEUROSCI.0861-19.2020 32001610 PMC7083284

[B5] Castelhano MS, Pereira EJ (2018) The influence of scene context on parafoveal processing of objects. Q J Exp Psychol 71:229–240. 10.1080/17470218.2017.1310263 28429648 PMC6154298

[B6] Cavanagh P, Hunt AR, Afraz A, Rolfs M (2010) Visual stability based on remapping of attention pointers. Trends Cogn Sci 14:147–153. 10.1016/j.tics.2010.01.007 20189870 PMC2847621

[B7] Cichy RM, Pantazis D, Oliva A (2014) Resolving human object recognition in space and time. Nat Neurosci 17:455–462. 10.1038/nn.3635 24464044 PMC4261693

[B8] Cimminella F, Sala SD, Coco MI (2020) Extra-foveal processing of object semantics guides early overt attention during visual search. Atten Percept Psychophys 82:655–670. 10.3758/s13414-019-01906-1 31792893 PMC7246246

[B9] Coco MI, Nuthmann A, Dimigen O (2020) Fixation-related brain potentials during semantic integration of object–scene information. J Cogn Neurosci 32:571–589. 10.1162/jocn_a_01504 31765602

[B10] Cortes C, Vapnik V (1995) Support-vector networks. Mach Learn 20:273–297. 10.1007/BF00994018

[B11] De Lissa P, McArthur G, Hawelka S, Palermo R, Mahajan Y, Degno F, Hutzler F (2019) Peripheral preview abolishes N170 face-sensitivity at fixation: using fixation-related potentials to investigate dynamic face processing. Vis Cogn 27:740–759. 10.1080/13506285.2019.1676855

[B12] Edwards G, VanRullen R, Cavanagh P (2018) Decoding trans-saccadic memory. J Neurosci 38:1114–1123. 10.1523/JNEUROSCI.0854-17.2017 29263239 PMC6596263

[B13] Ehinger BV, König P, Ossandón JP (2015) Predictions of visual content across eye movements and their modulation by inferred information. J Neurosci 35:7403–7413. 10.1523/JNEUROSCI.5114-14.2015 25972169 PMC6705440

[B14] Fabius JH, Fracasso A, Acunzo DJ, Van der Stigchel S, Melcher D (2020) Low-level visual information is maintained across saccades, allowing for a postsaccadic handoff between visual areas. J Neurosci 40:9476–9486. 10.1523/JNEUROSCI.1169-20.2020 33115930 PMC7724139

[B15] Ferrante O, Liu L, Minarik T, Gorska U, Ghafari T, Luo H, Jensen O (2022) FLUX: a pipeline for MEG analysis. Neuroimage 253:1–9. 10.1016/j.neuroimage.2022.119047 35276363 PMC9127391

[B16] Ganmor E, Landy MS, Simoncelli EP (2015) Near-optimal integration of orientation information across saccades. J Vis 15:1–12. 10.1167/15.16.8 26650193 PMC5079706

[B17] Gramfort A, et al. (2013) MEG and EEG data analysis with MNE-python. Front Neurosci 7:1–13. 10.3389/fnins.2013.00267 24431986 PMC3872725

[B18] Grootswagers T, Wardle SG, Carlson TA (2017) Decoding dynamic brain patterns from evoked responses: a tutorial on multivariate pattern analysis applied to time series neuroimaging data. J Cogn Neurosci 29:677–697. 10.1162/jocn_a_01068 27779910

[B19] Hebart MN, Dickter AH, Kidder A, Kwok WY, Corriveau A, Van Wicklin C, Baker CI (2019) THINGS: a database of 1,854 object concepts and more than 26,000 naturalistic object images. PLoS One 14:1–24. 10.1371/journal.pone.0223792 31613926 PMC6793944

[B20] Henderson JM (1992) Identifying objects across saccades: effects of extrafoveal preview and flanker object context. J Exp Psychol Learn Mem Cogn 18:521–530. 10.1037/0278-7393.18.3.521 1534353

[B21] Henderson JM, Pollatsek A, Rayner K (1987) Effects of foveal priming and extrafoveal preview on object identification. J Exp Psychol Hum Percept Perform 13:449–463. 10.1037/0096-1523.13.3.449 2958593

[B22] Henderson JM, Pollatsek A, Rayner K (1989) Covert visual attention and extrafoveal information use during object identification. Percept Psychophys 45:196–208. 10.3758/BF03210697 2710617

[B23] Henderson JM, Weeks PA Jr, Hollingworth A (1999) The effects of semantic consistency on eye movements during complex scene viewing. J Exp Psychol Hum Percept Perform 25:210–228. 10.1037/0096-1523.25.1.210

[B24] Herwig A (2015) Transsaccadic integration and perceptual continuity. J Vis 15:1–6. 10.1167/15.16.7 26650192

[B25] Huber-Huber C, Buonocore A, Dimigen O, Hickey C, Melcher D (2019) The peripheral preview effect with faces: combined EEG and eye-tracking suggests multiple stages of trans-saccadic predictive and non-predictive processing. Neuroimage 200:344–362. 10.1016/j.neuroimage.2019.06.059 31260837

[B26] Huber-Huber C, Buonocore A, Melcher D (2021) The extrafoveal preview paradigm as a measure of predictive, active sampling in visual perception. J Vis 21:1–23. 10.1167/jov.21.7.12 34283203 PMC8300052

[B27] Hung CP, Kreiman G, Poggio T, DiCarlo JJ (2005) Fast readout of object identity from macaque inferior temporal cortex. Science 310:863–866. 10.1126/science.1117593 16272124

[B28] Hunter JD (2007) Matplotlib: a 2D graphics environment. Comput Sci Eng 9:90–95. 10.1109/MCSE.2007.55

[B29] Isik L, Meyers EM, Leibo JZ, Poggio T (2014) The dynamics of invariant object recognition in the human visual system. J Neurophysiol 111:91–102. 10.1152/jn.00394.2013 24089402 PMC4280161

[B30] Jensen O, Pan Y, Frisson S, Wang L (2021) An oscillatory pipelining mechanism supporting previewing during visual exploration and reading. Trends Cogn Sci 25:1033–1044. 10.1016/j.tics.2021.08.008 34544653 PMC7615059

[B31] Kandemir G, Olivers C (2024) Comparing neural correlates of memory encoding and maintenance for foveal and peripheral stimuli. J Cogn Neurosci 36:1807–1826. 10.1162/jocn_a_02203 38940724 PMC11324249

[B32] King JR, Dehaene S (2014) Characterizing the dynamics of mental representations: the temporal generalization method. Trends Cogn Sci 18:203–210. 10.1016/j.tics.2014.01.002 24593982 PMC5635958

[B33] Kong G, Kroell LM, Schneegans S, Aagten-Murphy D, Bays PM (2021) Transsaccadic integration relies on a limited memory resource. J Vis 21:1–12. 10.1167/jov.21.5.24 34019621 PMC8142717

[B34] LaPointe MR, Milliken B (2016) Semantically incongruent objects attract eye gaze when viewing scenes for change. Vis Cogn 24:63–77. 10.1080/13506285.2016.1185070

[B35] Ludwig CJ, Davies JR, Eckstein MP (2014) Foveal analysis and peripheral selection during active visual sampling. Proc Natl Acad Sci U S A 111:E291–E299. 10.1073/pnas.1313553111 24385588 PMC3896144

[B36] Maris E (2012) Statistical testing in electrophysiological studies. Psychophysiology 49:549–565. 10.1111/j.1469-8986.2011.01320.x 22176204

[B37] Melcher D, Colby CL (2008) Trans-saccadic perception. Trends Cogn Sci 12:466–473. 10.1016/j.tics.2008.09.003 18951831

[B38] Morgan JL, Meyer AS (2005) Processing of extrafoveal objects during multiple-object naming. J Exp Psychol Learn Mem Cogn 31:428–442. 10.1037/0278-7393.31.3.428 15910129

[B39] Nichols TE, Holmes AP (2002) Nonparametric permutation tests for functional neuroimaging: a primer with examples. Hum Brain Mapp 15:1–25. 10.1002/hbm.1058 11747097 PMC6871862

[B40] Nuthmann A, De Groo F, Huettig F, Olivers CN (2019) Extrafoveal attentional capture by object semantics. PLoS One 14:1–19. 10.1371/journal.pone.0217051 31120948 PMC6532879

[B41] Otero-Millan J, Troncoso XG, Macknik SL, Serrano-Pedraza I, Martinez-Conde S (2008) Saccades and microsaccades during visual fixation, exploration, and search: foundations for a common saccadic generator. J Vis 8:1–18. 10.1167/8.14.21 19146322

[B42] Paeye C, Collins T, Cavanagh P (2017) Transsaccadic perceptual fusion. J Vis 17:1–11. 10.1167/17.1.14 28114484

[B43] Pan Y, Frisson S, Jensen O (2021) Neural evidence for lexical parafoveal processing. Nat Commun 12:1–9. 10.1038/s41467-020-20314-w 34475391 PMC8413448

[B44] Pedregosa F, et al. (2011) Scikit-learn: machine learning in Python. J Mach Learn Res 12:2825–2830.

[B45] Pollatsek A, Rayner K, Henderson JM (1990) Role of spatial location in integration of pictorial information across saccades. J Exp Psychol Hum Percept Perform 16:199–210. 10.1037/0096-1523.16.1.199 2137518

[B46] Prasad S, Galetta SL (2011) Anatomy and physiology of the afferent visual system. Handb Clin Neurol 102:3–19. 10.1016/B978-0-444-52903-9.00007-8 21601061

[B47] Ramík DM, Sabourin C, Moreno R, Madani K (2014) A machine learning based intelligent vision system for autonomous object detection and recognition. Appl Intell 40:358–375. 10.1007/s10489-013-0461-5

[B48] Rayner K (1998) Eye movements in reading and information processing: 20 years of research. Psychol Bull 124:372–422. 10.1037/0033-2909.124.3.372 9849112

[B49] Reichle ED, Liversedge SP, Pollatsek A, Rayner K (2009) Encoding multiple words simultaneously in reading is implausible. Trends Cogn Sci 13:115–119. 10.1016/j.tics.2008.12.002 19223223

[B50] Rohrbacker N (2009) Analysis of electroencephalogram data using time-delay embeddings to reconstruct phase space. Dynamics at the Horsetooth 1:1–11.

[B51] Schotter ER, Ferreira VS, Rayner K (2013) Parallel object activation and attentional gating of information: evidence from eye movements in the multiple object naming paradigm. J Exp Psychol Learn Mem Cogn 39:365–374. 10.1037/a0028646 22612163 PMC3670605

[B52] Skaramagkas V, Giannakakis G, Ktistakis E, Manousos D, Karatzanis I, Tachos NS, Tripoliti E, Marias K, Fotiadis DI, Tsiknakis M (2021) Review of eye tracking metrics involved in emotional and cognitive processes. IEEE Rev Biomed Eng 16:260–277. 10.1109/RBME.2021.3066072 33729950

[B53] Snell J, Grainger J (2019) Readers are parallel processors. Trends Cogn Sci 23:537–546. 10.1016/j.tics.2019.04.006 31138515

[B54] Stewart EE, Schütz AC (2018) Optimal trans-saccadic integration relies on visual working memory. Vision Res 153:70–81. 10.1016/j.visres.2018.10.002 30312623 PMC6241852

[B55] Vallat R (2018) Pingouin: statistics in Python. J Open Source Softw 3:1. 10.21105/joss.01026

[B56] Van der Linden L, Van de Putte E, Woumans E, Duyck W, Szmalec A (2018) Does extreme language control training improve cognitive control? A comparison of professional interpreters, L2 teachers and monolinguals. Front Psychol 9:1998. 10.3389/fpsyg.2018.01998 30405488 PMC6206226

[B57] Virtanen P, et al. (2020) Scipy 1.0: fundamental algorithms for scientific computing in Python. Nat Methods 17:261–272. 10.1038/s41592-019-0686-2 32015543 PMC7056644

[B58] Wäldchen J, Mäder P (2018) Machine learning for image based species identification. Methods Ecol Evol 9:2216–2225. 10.1111/2041-210X.13075

[B59] White AL, Boynton GM, Yeatman JD (2019) You can’t recognize two words simultaneously. Trends Cogn Sci 23:812–814. 10.1016/j.tics.2019.07.001 31477387 PMC8906166

[B60] Wijdenes LO, Marshall L, Bays PM (2015) Evidence for optimal integration of visual feature representations across saccades. J Neurosci 35:10146–10153. 10.1523/JNEUROSCI.1040-15.2015 26180191 PMC4502255

[B61] Winkler AM, Ridgway GR, Webster MA, Smith SM, Nichols TE (2014) Permutation inference for the general linear model. Neuroimage 92:381–397. 10.1016/j.neuroimage.2014.01.060 24530839 PMC4010955

[B62] Wolf C, Schütz AC (2015) Trans-saccadic integration of peripheral and foveal feature information is close to optimal. J Vis 15:1–18. 10.1167/15.16.1 26624936

[B63] Yarbus AL (1967) Eye movements and vision. New York: Springer. 10.1007/978-1-4899-5379-7

